# NUTRIC score in oncologic patients

**DOI:** 10.1186/cc14478

**Published:** 2015-03-16

**Authors:** A Patrão, L Bei, F Coelho

**Affiliations:** 1 Instituto Português de Oncologia - Porto, Portugal

## Introduction

The NUTRIC score is a tool designed to quantify the risk of critically ill patients developing adverse events that may be modified by aggressive nutrition therapy, in the general population of an ICU. Cancer patients are more prone to be at nutritional risk due to the disease and treatment complications. Our aim was to characterize NUTRIC score behavior in the population of patients admitted to an oncologic ICU.

## Methods

Between January and June 2014 we applied the NUTRIC score to all patients, age >18 years, without cerebral death criteria and with a length of stay (LOS) >72 hours. Data were collected and analyzed using SPSS v20.0. To evaluate the impact on mortality we used logistic regression.

## Results

Sixty-nine patients were included, 23 women (33.3%) and 46 men (66.7%). Most patients were aged between 50 and 75 years (72.5%) and had normal range weight 58% (*n *= 40). The mean LOS was 11.56 (minimum: 3 to maximum: 69). The most common motive for admission was sepsis (7.7%, *n *= 26). APACHE II score was above 15 in 77% of the patients (*n *= 53) and SOFA score was superior to 6 in 56.5% (*n *= 30). The NUTRIC score was low risk in 42% (*n *= 29) of the patients and high in 58% (*n *= 40). Twenty-eight-day mortality was 26.1% (*n *= 18). A high NUTRIC score corresponded to a 22-fold increased odds of dying in the first 28 days (*P *< 0.001). Both APACHE II and SOFA were mortality predictors alone, with an increase of 1 point in APACHE score corresponding to an increase of 14% (*P *= 0.002) and an increase in 1 point in SOFA corresponding to an increase in the odds of being dead at 28 days (*P *= 0.002). Body mass index, age, number of comorbidities, and days in the ICU did not correlate with mortality.

## Conclusion

The NUTRIC score is a good tool in cancer patients to predict 28-day mortality. Nevertheless, the only compounds of the NUTRIC score that correlated independently with mortality were APACHE II and SOFA scores. Further investigation towards the inclusion of other categories such as tumor staging and the type of tumor could be useful to develop a specific prognostic tool for this population.

